# NURSING HOME ADMISSION FROM RESIDENTIAL CARE FACILITY: NATIONAL HEALTH AGING TRENDS STUDY (NHATS) 2011–2019

**DOI:** 10.1093/geroni/igae098.2997

**Published:** 2024-12-31

**Authors:** Jung Yoen Son, Deanna J Marriott, Laura Struble, Chen Weiyun, Janet Larson

**Affiliations:** University of Michigan, Ann Arbor, Michigan, United States; University of Michigan, Ann Arbor, Michigan, United States; University of Michigan, Ann Arbor, Michigan, United States; University of Michigan, Ann Arbor, Michigan, United States; University of Michigan, Ann Arbor, Michigan, United States

## Abstract

Limited studies have examined risk factors for nursing home admission among residents in residential care facilities (RCF), and prior research is limited by short follow-ups, which are insufficient to observe changes in health and nursing home admission. This study aimed to (1) identify baseline factors associated with transfers to a nursing home and (2) identify time-varying factors associated with transfer to a nursing home over 8 years, using a national dataset from the National Health Aging Trends Study (NHATS) collected between 2011 and 2019. We conducted Cox proportional hazards regression to examine candidate predictors (chronic conditions, hospitalization, sleep disturbances, mental health, physical performance, difficulties in activities of daily living (ADL), self-reported health, social and physical activity, and sociodemographic) associated with nursing home admission. Employing backward elimination, we built parsimonious final models for analysis. The analytic sample included 970 participants (mean age at baseline=85.7 years, 67.2% women, 83.1% White, mean length of stay at baseline=2.3 years) and 143 had transferred to nursing homes over 8 years. Those who had better physical performance at baseline (HR=0.83,CI=0.79-0.88) and were college educated (HR=0.58,CI=0.37-0.86) demonstrated a significantly lower risk for nursing home admission over 8 years. Residents who maintained physical activity (HR=0.56,CI=0.37-0.86), better physical performance (HR=0.87,CI=0.80-0.94), and fewer ADL difficulties (HR=1.13,CI=1.02-1.26) were at lower risk for nursing home admission over 8 years. Our findings can assist in identifying older adults in RCFs at risk of nursing home admission. Strategies to promote physical function and physical activity could be helpful to avoid/delay such admission.

